# Biomimetics inspired surfaces for drag reduction and oleophobicity/philicity

**DOI:** 10.3762/bjnano.2.9

**Published:** 2011-02-01

**Authors:** Bharat Bhushan

**Affiliations:** 1 Nanoprobe Laboratory for Bio- & Nanotechnology and Biomimetics (NLB²), The Ohio State University, 201 W. 19th Avenue, Columbus, OH 43210-1142, USA

**Keywords:** aquatic animals, biomimetics, drag, lotus plants, shark skin, superhydrophobicity, superoleophobicity

## Abstract

The emerging field of biomimetics allows one to mimic biology or nature to develop nanomaterials, nanodevices, and processes which provide desirable properties. Hierarchical structures with dimensions of features ranging from the macroscale to the nanoscale are extremely common in nature and possess properties of interest. There are a large number of objects including bacteria, plants, land and aquatic animals, and seashells with properties of commercial interest. Certain plant leaves, such as lotus (*Nelumbo nucifera*) leaves, are known to be superhydrophobic and self-cleaning due to the hierarchical surface roughness and presence of a wax layer. In addition to a self-cleaning effect, these surfaces with a high contact angle and low contact angle hysteresis also exhibit low adhesion and drag reduction for fluid flow. An aquatic animal, such as a shark, is another model from nature for the reduction of drag in fluid flow. The artificial surfaces inspired from the shark skin and lotus leaf have been created, and in this article the influence of structure on drag reduction efficiency is reviewed. Biomimetic-inspired oleophobic surfaces can be used to prevent contamination of the underwater parts of ships by biological and organic contaminants, including oil. The article also reviews the wetting behavior of oil droplets on various superoleophobic surfaces created in the lab.

## Introduction

Biologically inspired design, adaptation, or derivation from nature is referred to as ‘biomimetics.’ It means mimicking biology or nature. Nature has gone through evolution over the 3.8 billion years since life is estimated to have appeared on the Earth [1]. Nature has evolved objects with high performance using commonly found materials. These function on the macroscale to the nanoscale. The understanding of the functions provided by objects and processes found in nature can guide us to imitate and produce nanomaterials, nanodevices, and processes [2]. There are a large number of objects (bacteria, plants, land and aquatic animals, seashells etc.) with properties of commercial interest.

### Natural superhydrophobic, self-cleaning, low adhesion, and drag reduction surfaces

**Figure 1:**
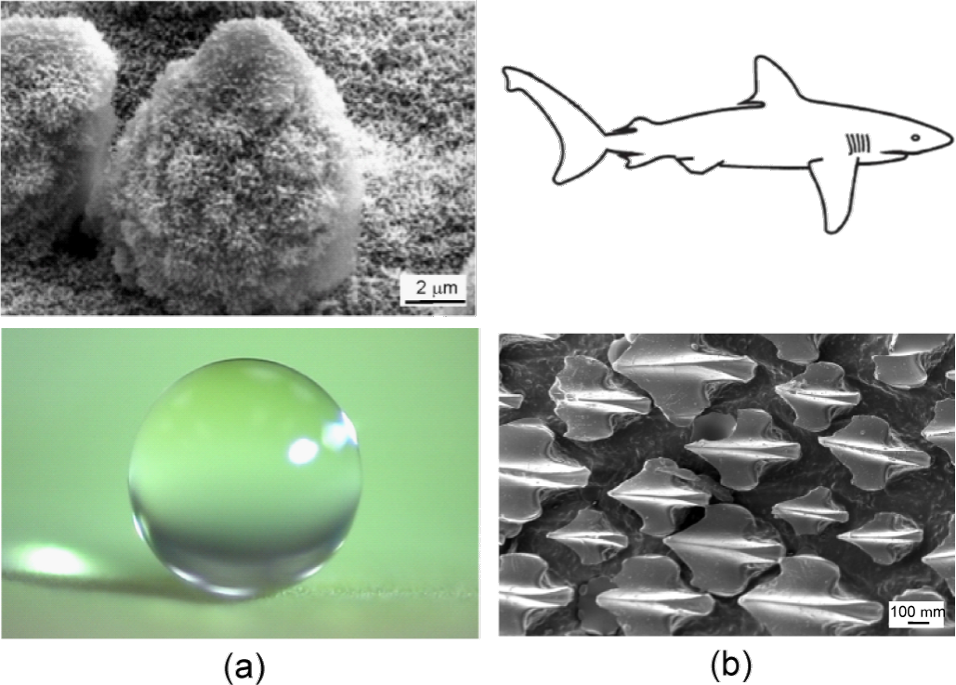
Two examples from nature: (a) Lotus effect [12], and (b) scale structure of shark reducing drag [21].

Drag reduction in fluid flow is of interest in various commercial applications. These include transportation vehicles and micro/nanofluidics based biosensor applications [3]. To reduce pressure drop and volume loss in micro/nanochannels used in micro/nanofluidics, it is desirable to minimize the drag force at the solid–liquid interface. A model surface for superhydrophobicity, self-cleaning and low adhesion is the leaves of water-repellent plants such as *Nelumbo nucifera* (lotus) [2,4–11]. The leaf surface is very rough due to so-called papillose epidermal cells, which form papillae or microasperities. In addition to the microscale roughness, the surface of the papillae is also rough, with nanoscale asperities composed of three-dimensional epicuticular waxes which are long chain hydrocarbons and hydrophobic. The waxes on lotus leaves exist as tubules [10–11]. Water droplets on these hierarchical structured surfaces readily sit on the apex of the nanostructures because air bubbles fill the valleys of the structure under the droplet (Figure 1a). Therefore, these leaves exhibit considerable superhydrophobicity. Static contact angle and contact angle hysteresis of a lotus leaf are about 164° and 3°, respectively [12–13]. The water droplets on the leaves remove any contaminant particles from their surfaces when they roll off, leading to self-cleaning [5] and show low adhesive force [14–16].

### Natural superoleophobic, self-cleaning, and drag reduction surfaces

A model surface for superoleophobicity and self-cleaning is provided by fish which are known to be well protected from contamination by oil pollution although they are wetted by water [15,17]. Fish scales have a hierarchical structure consisting of sector-like scales with diameters of 4–5 mm covered by papillae 100–300 μm in length and 30–40 µm in width [18]. Shark skin, which is a model from nature for a low drag surface, is covered by very small individual tooth-like scales called dermal denticles (little skin teeth), ribbed with longitudinal grooves (aligned parallel to the local flow direction of the water) (Figure 1b). These grooved scales reduce vortice formation present on a smooth surface, resulting in water moving efficiently over their surface [2,19–22]. The water surrounding these complex structures can lead to protection from marine fouling and play a role in defense against adhesion and growth of marine organisms, e.g., bacteria and algae [11,23]. If oil is present on the surfaces in air or water, surfaces are known to be oleophobic and may exhibit self-cleaning and anti-fouling properties. Many sea animals including fish and shark are known to be oleophobic under water. Superoleophobic surfaces can also reduce significant losses of residual fuel in fuel tanks and pipes [15,24].

### Roughness-induced superhydrophobicity, self-cleaning, low adhesion, and drag reduction

**Figure 2:**
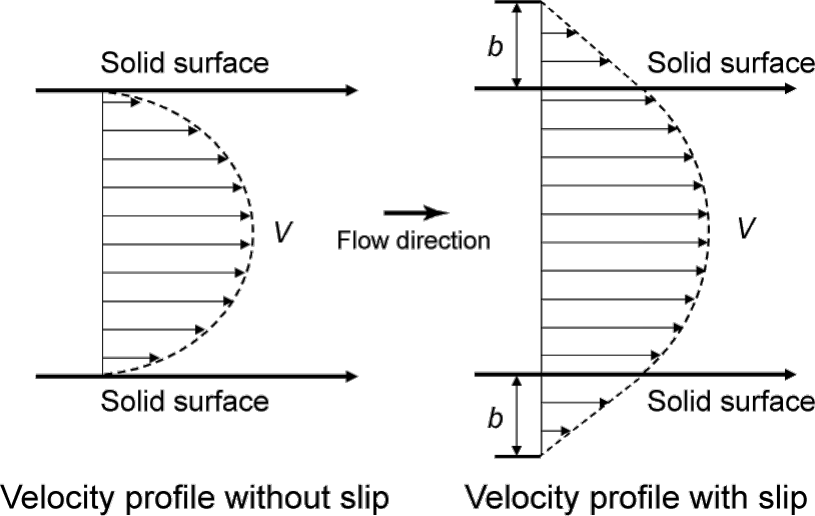
Schematic of velocity profiles of fluid flow without and with boundary slip. The definition of slip length *b* characterizes the degree of boundary slip at the solid–liquid interface. The arrows represent directions of fluid flow.

Jung and Bhushan [21] created artificial surfaces inspired by the lotus leaf and shark skin and studied the influence of structure on pressure drop and fluid drag. One of the basic properties of interest in fluid flow is slip. The relative velocity between a solid wall and liquid is believed to be zero at the solid–liquid interface, which is the so called no-slip boundary condition (Figure 2, left) [25–26]. However, for hydrophobic surfaces, fluid film exhibits a phenomenon known as slip, which means that the fluid velocity near the solid surface is not equal to the velocity of the solid surface (Figure 2, right) [27–33]. The degree of boundary slip at the solid–liquid interface is characterized by a slip length. The slip length *b* is defined as the length of the vertical intercept along the axis orthogonal to the interface when a tangent line is drawn along the velocity profile at the interface (Figure 2, right). Recent experiments with surface force apparatus (SFA) [34–36], atomic force microscopy (AFM) [32–33,37], and particle image velocimetry (PIV) [38] techniques have reported slip lengths on hydrophobic surfaces: No slip was observed on hydrophilic surfaces [34,36–40]. Theoretical studies [41–44] and experimental studies [33,45–47] suggest that the presence of nanobubbles at the solid-liquid interface is responsible for boundary slip on hydrophobic surfaces.

### Roughness-induced superoleophobicity

The surface tension of oil and organic liquids is lower than that of water, so to create a superoleophobic surface, the surface energy of the solid surface in air should be lower than that of oil. For underwater applications, if an oil droplet is placed on a solid surface in water, the solid–water–oil interface exists. The nature of oleophobicity/philicity of an oil droplet in water can be determined from the values of surface energies of various interfaces and contact angles of water and oil in air.

Many superoleophobic surfaces have been developed by modifying the surface chemistry with a coating of extreme low surface energy materials [20,48–54]. Tuteja et al. [54] showed that surface curvature, in conjunction with chemical composition and roughened texture, can be used for liquids with low surface tension, including alkanes such as decane and octane. Liu et al. [18] performed experiments in a solid-water-oil interface. They found that hydrophilic and oleophilic surfaces (solid–air–water interface and solid–air–oil interface) can switch to an oleophobic surface in water (solid–water–oil interface). As a result, oil contaminants are washed away when immersed in water. This effect can be employed for underwater oleophobicity and self-cleaning that can be used against marine ship fouling [17]. Jung and Bhushan [20] proposed a model for predicting the oleophobic/philic nature of surfaces and showed how the water and oil droplets in three phase interfaces influence the wetting behavior on micropatterned surfaces with varying pitch values as well as the shark skin replica as an example from an aquatic animal.

### Article objective

This article reviews drag data on artificial surfaces inspired from shark skin and lotus leaf. Oleophobic and self-cleaning surfaces inspired from aquatic animals are then discussed.

## Fabrication and Characterization of Biomimetic Structures for Fluid Drag Reduction

In this section, we discuss drag reduction efficiency on biomimetic structured surfaces in channels.

### Experimental techniques

**Figure 3:**
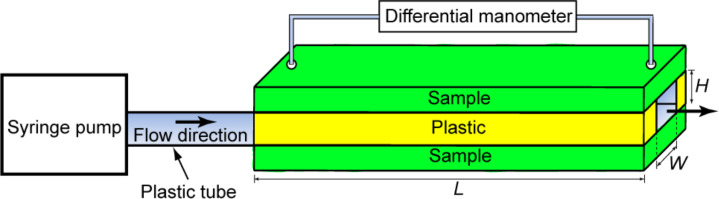
Schematic of the experimental flow channel connected with a differential manometer. The thickness, width, and length of the channel are *H*, *W*, and *L*, respectively.

For the measurement of pressure drop using water and air flows, an experimental flow channel with a rectangular channel was designed and fabricated as shown in Figure 3 [21]. The fabricated surfaces were used for the upper and lower walls of the flow channel. Two pieces of plastic were glued between the upper and lower samples and at each end to prevent flow leak. For the measurement of pressure drop, the upper sample had two opening holes connected with a differential manometer (Model A 1000-13, Differential Pressure Plus Inc., USA). The thickness, width, and length of the resulting channel are designated as *H*, *W*, and *L*, respectively.

The inlet and outlet ports were machined and connected with plastic tubes. To introduce water into the channel in laminar flow, a syringe pump (Model NE-300, New Era Pump Systems Inc., USA) was used at a range of flow rates between 50 μL/s and 400 μL/s (a range of flow velocity between 0.03 m/s and 0.23 m/s). The Reynolds number of the flow applied by the syringe pump was less than 300, which is the laminar flow. To create a turbulent flow, a larger flow rate is needed that cannot be accomplished with the syringe pump. To accomplish high fluid flow, a separate plastic chamber filled with a measured amount of water was used to allow flow through the channel under the force of gravity. By measuring the amount of water and time for the water to flow from a starting fluid level to an end fluid level, the Reynolds number was calculated as 4200 (flow velocity of 3.8 m/s), which indicates that the flow is turbulent using this setup. In order to make air flow a laboratory air outlet was connected to the channel. A flowmeter (Model FL-1478-G, Omega Engineering, Inc., USA) was used to measure the air flow rate between laboratory air outlet and channel. For the experimental measurements of air flow, the calculated range of Reynolds number was between 200 and 4600, which indicates both laminar and turbulent flows [21].

### Model for calculation of pressure drop and slip length

The pressure drop Δ*p* of an incompressible fluid flow between two points along the channel of thickness *H*, width *W*, and length *L* for a hydrophilic flat surface can be calculated by [55]

[1]
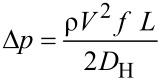


where ρ is the fluid density, *V* is the flow velocity obtained from flow rate *Q* divided by cross section area of the channel, and *f* is the friction factor which specifies the loss in pressure required to impel a flow over the surface or through the channel. The friction factor is generally a function of Reynolds number, surface roughness, and the geometry of the surface. *D*_H_ is the hydraulic diameter which is proportional to four times the flow area divided by the perimeter of the surface containing the flow. For the rectangular channel, the hydraulic diameter is

[2]
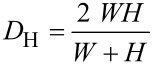


The friction factor for laminar flow is inversely proportional to the Reynolds number *Re* as [55]

[3]
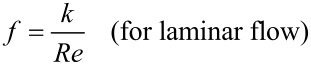


[4]
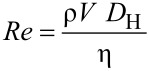


where η is the dynamic fluid viscosity. The Reynolds number can be used to determine whether the fluid flow will be within the laminar, turbulent, or transitional flow regimes. Since the Reynolds number is proportional to flow velocity, the pressure drop in laminar flow increases with flow velocity. *k* is the friction coefficient which can be found by the solution of Poisson’s equation over the cross section as [55]

[5]
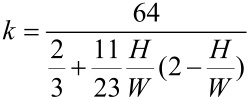


From Equation 5, the friction coefficient is dependent only on the shapes of the cross section and independent of the surface roughness.

To improve the calculation of the friction factor for turbulent flow in a rectangular channel, Jones [56] developed an improved equivalent diameter, *D*_e_ = 64*D*_H_/*k*, thus the friction factor for turbulent flow can be modified as

[6]
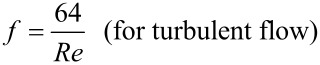


Next, we present an analysis to calculate slip length in laminar flow. Using the Navier slip boundary condition, the slip length *b* of the two infinite parallel and smooth plates can be expressed as [45,55]

[7]
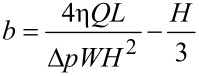


For a rectangular channel, the slip length would have the following general form [45]

[8]
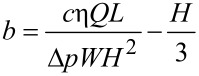


where *c* is a constant which must be obtained empirically. In order to obtain the constant, pressure drop measurements on a hydrophilic channel must be made. Equation 8 is then fitted under the assumption of zero slip length with the measured pressure drop data to obtain *c* and was equal to 5 for the channel (*H* = 0.7 mm, *W* = 2.5 mm, *L* = 60 mm) used in this study. This equation was now used to calculate the slip length for hydrophobic surfaces [21].

### Fabrication and characterization of biomimetic structures

A shark (*Squalus acanthias*, L. Squalidae) was used for creating a shark skin replica [21]. A shark is an aquatic animal, and its skin is permanently exposed to contamination from marine organisms, e.g., bacteria and algae. The shark was conserved in FAA (formaldehyde/acetic acid/ethanol) solution. The detailed structure varies from one location to another for the shark. The scales are present over most of the shark’s body. To create a replica, the right front of shark body was selected. Before replicating the conserved shark skin, the selected area was first cleaned with acetone and then washed with deionized water. This process was repeated twice. The cleaned skin was placed in air for 1 hour for drying. For the negative replica, a polyvinylsiloxane dental wax was applied via a dispenser on the upper side of the shark skin and immediately pressed down with a glass plate. After complete hardening of the molding mass (at room temperature 3–5 minutes), the master surface and the mold (negative) were separated. The first negative replica was made only to remove any remaining contaminations from the shark surface by embedding the dirt into the replica material. A second and third replica of the same area was made to obtain negatives without contamination. For the positive replica, a liquid epoxy resin was used in the molding process.

To simulate a shark skin structure, a rib-patterned surface was created using a FlashCut CNC milling machine [21]. Bechert et al. [57] and Dean and Bhushan [22] have reported that optimal groove depth for the rib surface should be about half of the lateral rib spacing for low drag. In the rib pattern design selected here, multiple stacks of ribs oriented along an axis were fabricated. For the fabrication, first a model of a rib-patterned surface was designed in SolidWorks, and then the code for the rib’s height, width, spacing and lengths, and channel dimensions was written with FeatureCAM in order to fabricate structures using the CNC milling machine. An acrylic resin was clamped onto the table of the CNC mill, and a fly cutter was used to make the top of the surface flat. The code was opened with FlashCut CNC and then the rib patterns were milled using an endmill with 130 μm bit.

**Figure 4:**
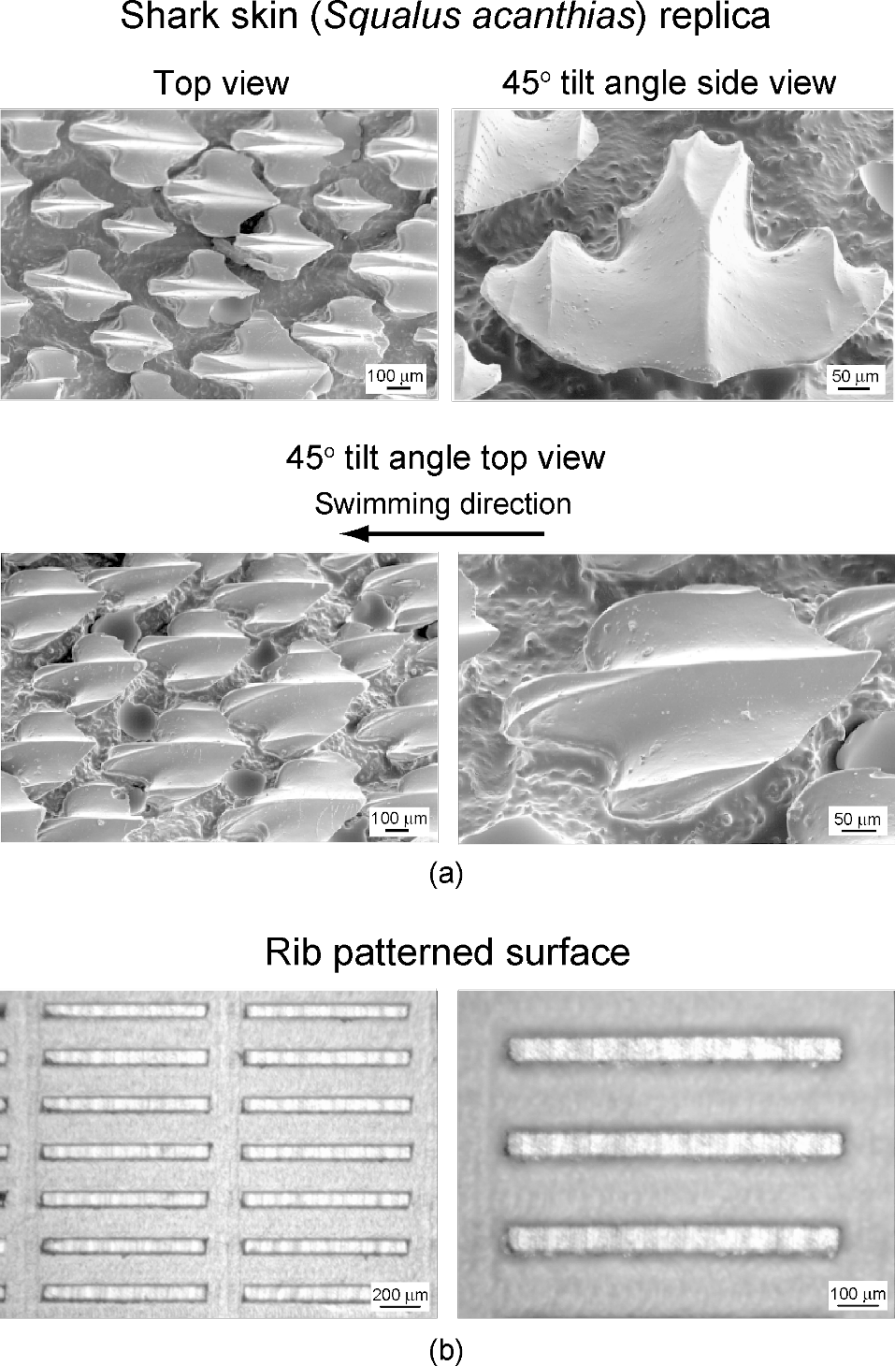
(a) SEM micrographs taken at top view, 45° tilt angle side view and 45° tilt angle top view, show shark skin (*Squalus acanthias*) replica. The shark skin replica shows only three ribs on each scale. (b) optical microscope images (shown at two magnifications) show the rib-patterned surface fabricated as a model of artificial shark skin surfaces [21].

Figure 4a shows the scanning electron microscope (SEM) micrographs of the shark skin (*Squalus acanthias*) replica taken at a top view, a 45° tilt angle side view, and a 45° tilt angle top view. The shark skin replica shows that scales are lifted up at the end, and there are only three ribs on each scale. It is clearly visible that the V-shaped riblets’ height varies between 200 and 500 µm, and their space varies between 100 and 300 μm. The ribs are oriented nearly parallel to swimming direction of the shark. Figure 4b shows the optical microscope images of the rib-patterned surface fabricated as a model of artificial shark skin surfaces. The height, width, and length of the created ribs were 90, 38, and 850 μm, respectively. The spacing between the ribs was 180 µm.

To investigate drag reduction efficiency on the surfaces with superhydrophobicity, self-cleaning, and low adhesion described earlier, Jung and Bhushan [21] used nano-, micro-, and hierarchical structures [12–13]. Microstructures were fabricated using a two step molding process (soft lithography). A microstructured Si surface with pillars of 14 μm diameter and 30 μm height with 23 μm pitch fabricated by photolithography was used as a master template. A negative replica of the template was generated by applying a polyvinylsiloxane dental wax (President Light Body^®^ Gel, ISO 4823, Polyvinylsiloxan (PLB), Coltene Whaledent, Hamburg, Germany), via a dispenser on the surface and immediately pressing down with a glass plate. After complete hardening of the molding mass (3–5 minutes at room temperature), the silicon master surface and the mold (negative) were separated. After a relaxation time of 30 minutes for the molding material, the negative replicas were filled with a liquid epoxy resin (Epoxydharz L^®^, No. 236349, Conrad Electronics, Hirschau, Germany) with hardener (Harter S, Nr 236365, Conrad Electronics, Hirschau, Germany). Specimens with microstructures were immediately transferred into a vacuum chamber at 750 mTorr (100 Pa) pressure for 10 seconds to remove trapped air and to increase the resin infiltration through the structures. After hardening at room temperature (24 h at 22 °C), the positive replica was separated from the negative replica. To generate several replicas the second step of replication was repeated twenty times for each surface type.

**Figure 5:**
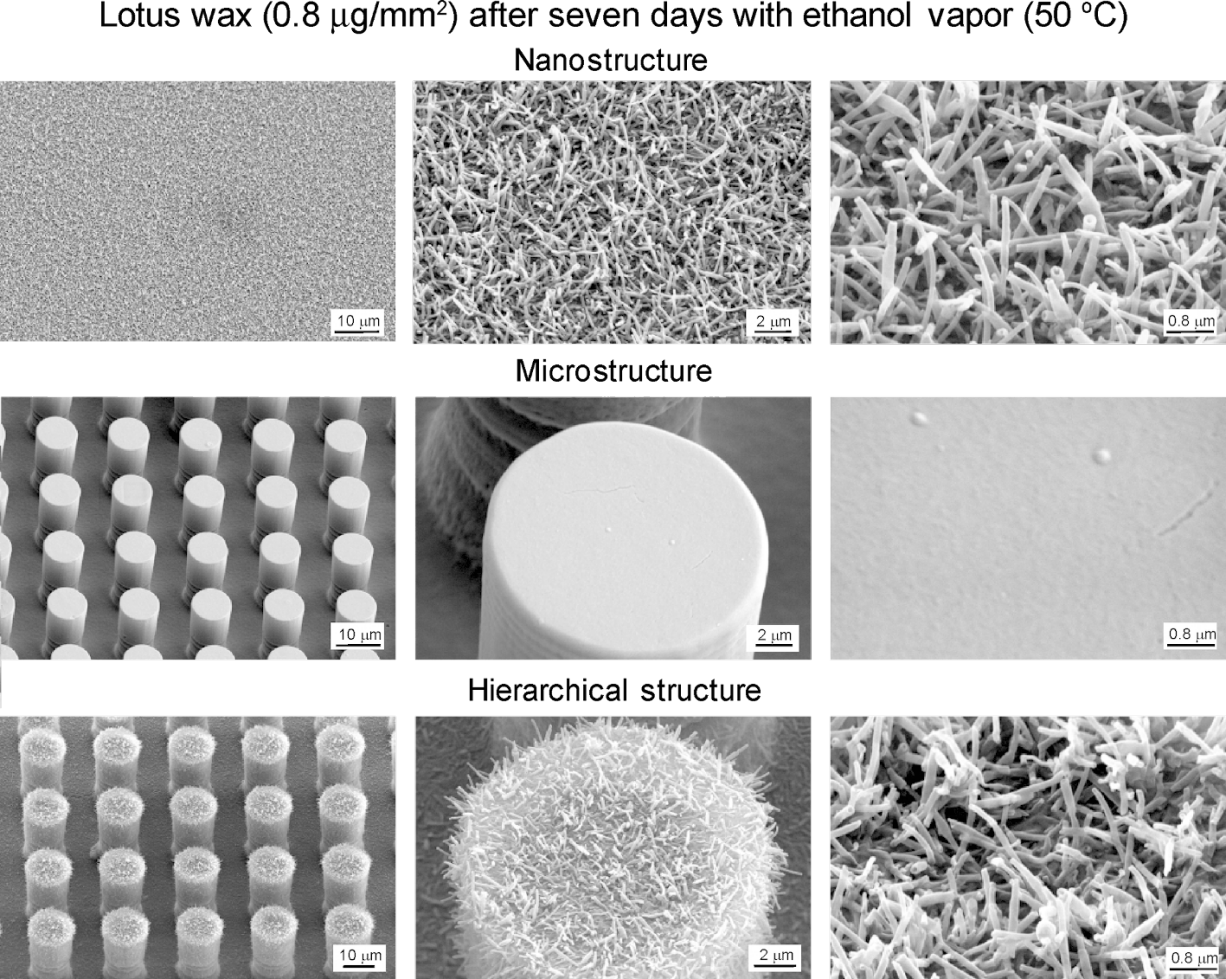
SEM micrographs taken at 45º tilt angle (shown using three magnifications) of nanostructure on flat replica, microstructures, and hierarchical structure. Nano and hierarchical structures coated with 0.8 μg/mm^2^ of Lotus wax after storage for seven days at 50 °C with ethanol vapor. Flat epoxy resin and microstructure were covered with flat Lotus wax [21].

Nanostructures were created by self-assembly of plant wax deposited by thermal evaporation [12–13]. Tubule forming wax, which was isolated from a leaf of *Nelumbo nucifera,* in the following referred to as Lotus, was used to create tubule structures. Lotus wax with 0.8 µg/mm^2^ was deposited on the specimen surfaces by thermal evaporation. The specimens with Lotus wax were exposed to ethanol vapor for three days at 50 °C, and then left in the oven at 50 °C for seven days in total. Hierarchical structures were fabricated by creating of nanostructures on top of microstructured surfaces, as described above. Flat epoxy resin and microstructure were covered with flat Lotus wax. Flat thin wax layer was made by melting the deposited wax (3 min at 120 °C) and subsequent rapid cooling of the specimen to 5 °C. Then the specimens were stored for seven days at 21 °C in a desiccator. The fast cooling of the wax prevents the formation of nanostructure roughness. Figure 5 shows the SEM micrographs of nanostructure on flat replica, microstructures, and hierarchical structure. SEM micrographs show an overview (left column), a detail in higher magnification (middle column), and a large magnification of the created flat wax layers and tubules nanostructures (right column).

Table 1 summarizes the static contact angle and contact angle hysteresis measured on shark skin replica, rib-patterned surface, and the structured surfaces with Lotus wax. The shark skin replica had a static contact angle of 89° and a contact angle hysteresis of 66° for a water droplet. For acrylic resin material, a static contact angle of 82° was found for flat acrylic resin. Introduction of the rib patterns on the flat surface led to a much higher static contact angle of 146° and lower contact angle hysteresis of 43°. A static contact angle of 76° was found for flat epoxy resin. The microstructure (covered with a Lotus wax film) has a static contact angle of 160° but shows a much higher contact angle hysteresis of 27° than found in the hierarchical structure. Superhydrophobicity with a static contact angle of 167° and a contact angle hysteresis of 6° was also found in the nanostructured surface. Melting of the wax led to a flat surface with a flat wax film and a much lower static contact angle of 119° and higher contact angle hysteresis of 56°. The data of a flat Lotus wax film on a flat replica show that the Lotus wax by itself is hydrophobic. For the hierarchical structure, the highest static contact angles of 173° and lowest contact angle hysteresis of 1° were found. The recrystallized wax tubules are very similar to those of the original lotus leaf, but are 0.5 to 1 µm longer, and the static contact angle is higher, and the contact angle hysteresis is lower than reported for the original lotus leaf (static contact angle of 164° and contact angle hysteresis of 3°).

**Table 1:** Summary of the static contact angles and contact angle hysteresis measured on the various surfaces. Nanostructures and hierarchical structures were fabricated with 0.8 μg/mm^2^ of Lotus wax after storage at 50 °C with ethanol vapor. Flat epoxy resin and microstructure were covered with flat Lotus wax. The variations represent one standard deviation [21].

	Static contact angle (°)	Contact angle hysteresis (°)

(a) Epoxy resin		

Flat epoxy resin	76 ± 0.9	67 ± 2.9 (151^a^, 84^b^)
Flat with thin wax layer	119 ± 2.4	56 ± 3.2 (148^a^, 92^b^)
Nanostructure	167 ± 0.7	6 ± 1.1 (170^a^, 164^b^)
Microstructure	160 ± 1.8	27 ± 2.1 (169^a^, 142^b^)
Hierarchical structure	173 ± 0.8	1 ± 0.6 (174^a^, 173^b^)
Shark skin replica	89 ± 1.7	66 ± 3.4 (155^a^, 89^b^)

(b) Acrylic resin		

Flat acrylic resin	82 ± 1.8	71 ± 2.6 (122^a^, 51^b^)
Rib-patterned surface	146 ± 1.2	43 ± 1.2 (158^a^, 115^b^)

^a^advancing contact angle; ^b^receding contact angle.

**Figure 6:**
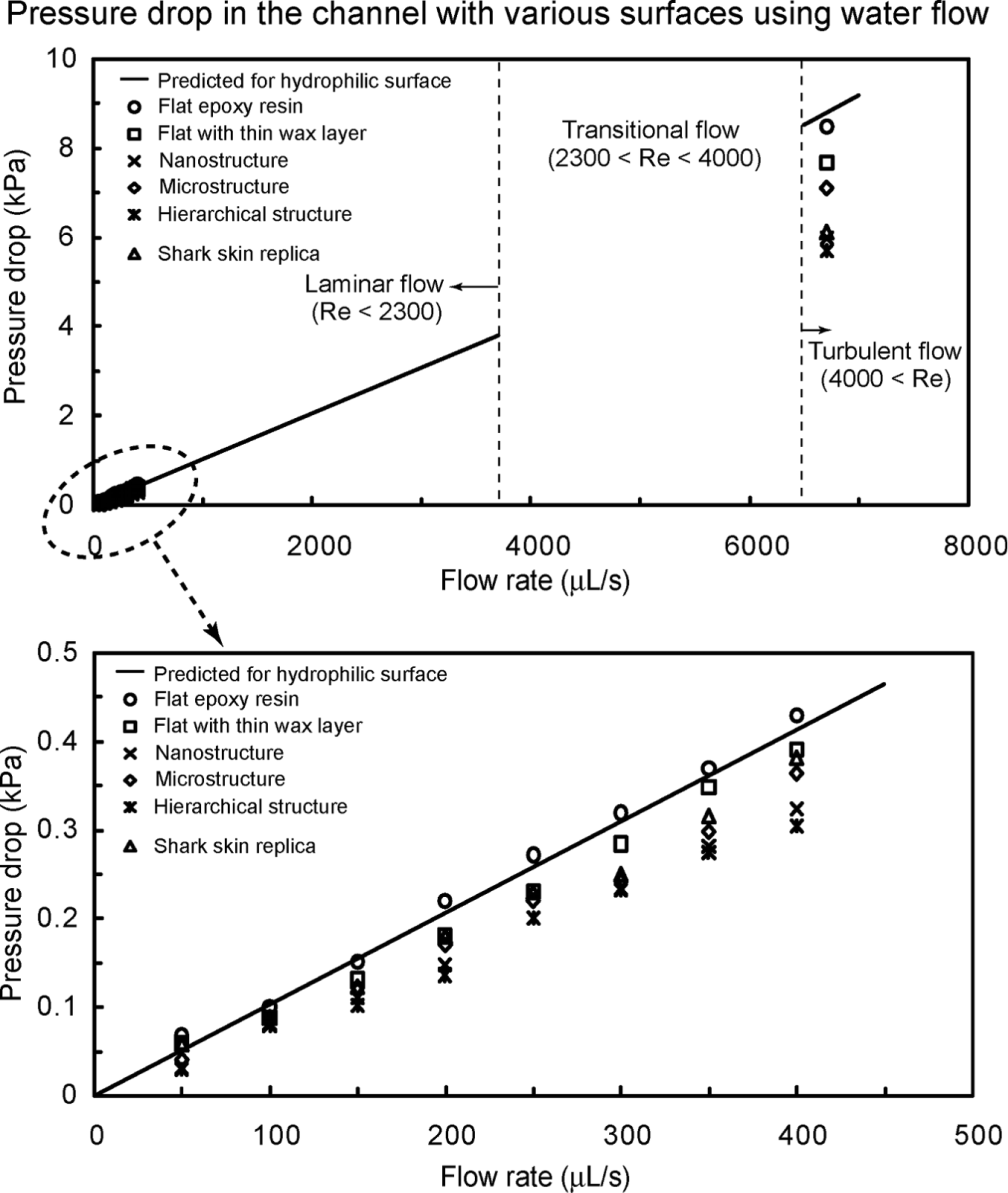
Pressure drop as a function of flow rate in the channel with various surfaces using water flow. The figure in the bottom is magnified in flow rate between 0 and 500 μL/s. Data are compared with predicted pressure drop values for a hydrophilic surface obtained using Equation 1 for laminar and turbulent flows (solid lines) [21].

**Pressure drop in the channel using water flow and calculated slip length.** To observe the fluid drag reduction in the channel using water flow, experiments on flat epoxy resin, flat with thin wax layer, nanostructure, microstructure, hierarchical structure, and shark skin replica were performed [21]. In Figure 3, the rectangular channels with these surfaces had thickness *H* = 0.7 mm, width *W* = 2.5 mm, and length *L* = 60 mm. For calculation of the pressure drop using Equation 1, the mass density ρ and viscosity η for water were taken to be 1000 kg/m^3^ and 0.001 Pa·s, respectively [58]. Figure 6 shows the pressure drop as a function of flow rate in the channel with various surfaces using water flow. The measured data are compared with the predicted pressure drop values for a hydrophilic surface obtained using Equation 1 for laminar and turbulent flows (solid lines). The figure in the bottom is magnified in flow rate between 0 and 500 µL/s. In both laminar and turbulent flows, the pressure drop increased linearly with flow rate for all samples. It was found that the pressure drop for the flat epoxy resin was similar to the value predicted by Equation 1, while structured surfaces had values lower than the predicted. As mentioned earlier and shown in Table 1, the introduction of roughness increases the hydrophobicity of the surfaces responsible for reduction in drag or pressure drop. The hierarchical structure with highest contact angle and lowest static contact angle hysteresis provided the lowest pressure drop. It is believed that air pockets inside the grooves underneath the fluid reduce the contact area between fluid and surface, resulting in lower pressure drop. These results indicate that superhydrophobicity can lead to drag reduction in fluid flow [21].

As shown in Figure 6, for shark skin replica, it was found that the pressure drop in laminar flow was higher than those of the nanostructure and hierarchical structure and the reduction of pressure drop was about 12% as compared to the theoretical pressure drop. However, in turbulent flow, the reduction of pressure drop was similar to those of nanostructure and hierarchical structure. Bechert et al. [19] showed that a turbulent boundary layer on the shark skin surface with ribs can help to reduce turbulent shear stress (also see Dean and Bhushan [22]). The results of experimental measurements on shark skin replica showed that a reduction of pressure drop was obtained up to 30% in turbulent flow. It can be concluded that the surfaces with ribs are more beneficial in providing drag reduction in turbulent flow than in laminar flow.

**Figure 7:**
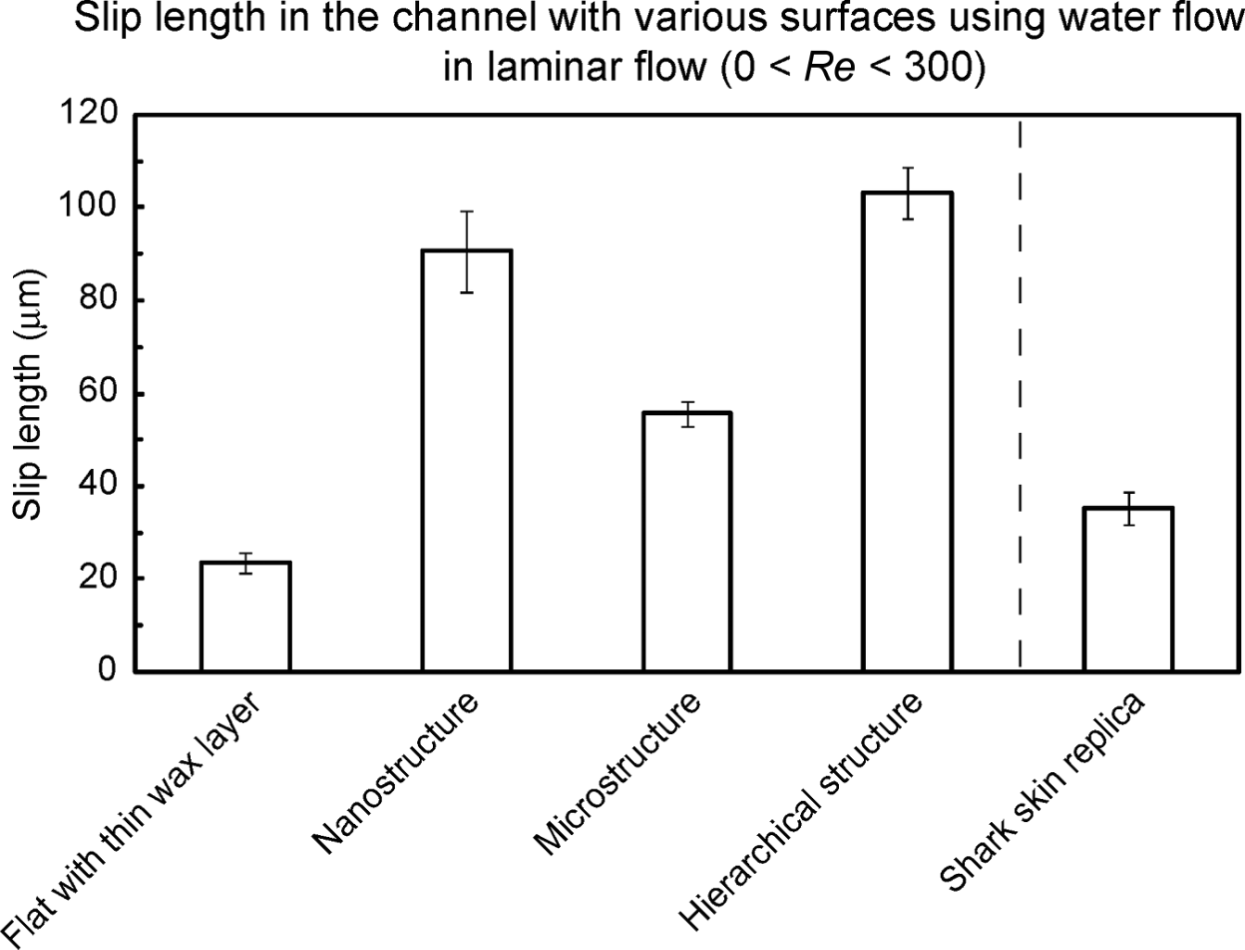
Bar chart showing the slip length in the channel with various surfaces using water flow in laminar flow (0 < *Re* < 300). The slip length was calculated using Equation 8 and the pressure drop measured on various surfaces. The error bars represent one standard deviation [21].

Based on the pressure drop data, the slip length on the surfaces with different wettabilities was calculated using Equation 8. For calculations, it was assumed that there is a no-slip boundary condition on flat epoxy resin as verified from the experiments [40]. Figure 7 shows the bar chart showing the slip length in the channel with various surfaces using water flow in laminar flow (0 < *Re* < 300). The average values of slip length on the surfaces were calculated over all the experimental flow rates. A slip length of 24 µm was found for the flat surface with thin wax layer. The microstructure (covered with a flat Lotus wax film) and shark skin replica had slip lengths of 56 and 35 µm, but the nanostructure and hierarchical structures show much higher slip lengths of 91 and 103 µm, respectively, which implies the boundary slip increases with increasing hydrophobicity of solid surfaces.

Slip length measurements have also been made on the nanoscale on hydrophilic and hydrophobic surfaces with various degrees of hydrophobicity using a dynamic AFM method [16,33]. Data on one hydrophilic, one hydrophobic, and one superhydrophobic surface are presented in Table 2. Mica was taken as the hydrophilic surface. Hydrophobic and superhydrophobic surfaces were fabricated by deposition of evaporated plant wax on smooth epoxy substrates following the procedure described earlier in this section. Hydrophilic surface was produced without any recrystallization (rather flat surface) whereas to produce a superhydrophobic surface, the wax was recrystallized to produce a tubular nanostructure [13]. The data presented in Table 2 shows increasing boundary slip from the hydrophobic surface to the superhydrophobic one. We note that slip length on the nanoscale is much lower than that on the macroscale reported in Figure 7. Zhu and Granick [59] have reported that the slip length increases from nanometer range to micrometer range as the flow rate increases.

**Table 2:** RMS roughness (AFM, scan size = 5 μm × 5 μm), static contact angle and slip length measured using tapping mode of three different surfaces [16,33].

Surfaces	RMS roughness (nm)	Static contact angle (°)	Slip length with tapping AFM (nm)

hydrophilic	0.20	~0	~0
hydrophobic	11.0	91 ± 2.0	43 ± 10
superhydrophobic	178	167 ± 0.7	236 ± 18

**Figure 8:**
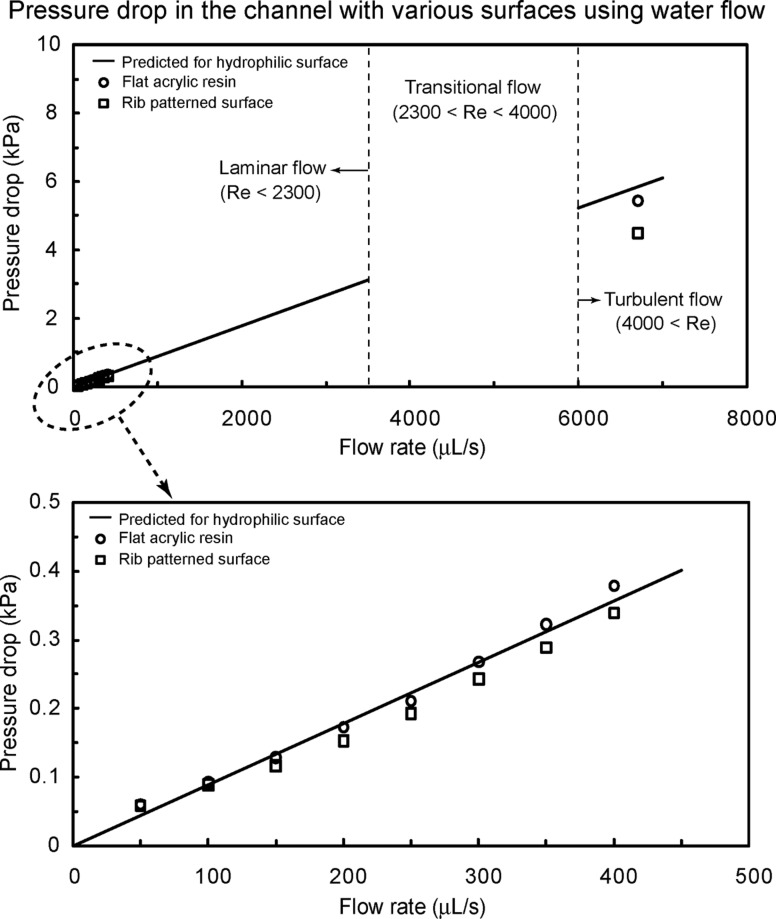
Pressure drop as a function of flow rate in the channel with flat acrylic resin and rib-patterned surface using water flow. The figure in the bottom is magnified for flow rate between 0 and 500 µL/s. Data are compared with predicted pressure drop values for a hydrophilic surface obtained using Equation 1 for laminar and turbulent flows (solid lines) [21].

The fluid drag measurements were also made on flat acrylic resin and rib-patterned surfaces fabricated as a model of artificial shark skin [21]. In Figure 3, the rectangular channels with these surfaces had thickness *H* = 1 mm, width *W* = 2 mm, and length *L* = 100 mm. Figure 8 shows the pressure drop as a function of flow rate in the channel using water flow. The measured data are compared with predicted pressure drop values for a hydrophilic surface obtained using Equation 1 for laminar and turbulent flows (solid lines). The figure at the bottom is magnified for flow rate between 0 and 500 μL/s. In laminar flow, it was found that the pressure drop increased linearly with flow rate and was similar to the value predicted by Equation 1. However, in turbulent flow, the reduction in pressure drop was up to 23% as compared to the theoretical pressure drop. This result shows a similar trend to that of the shark skin replica.

**Figure 9:**
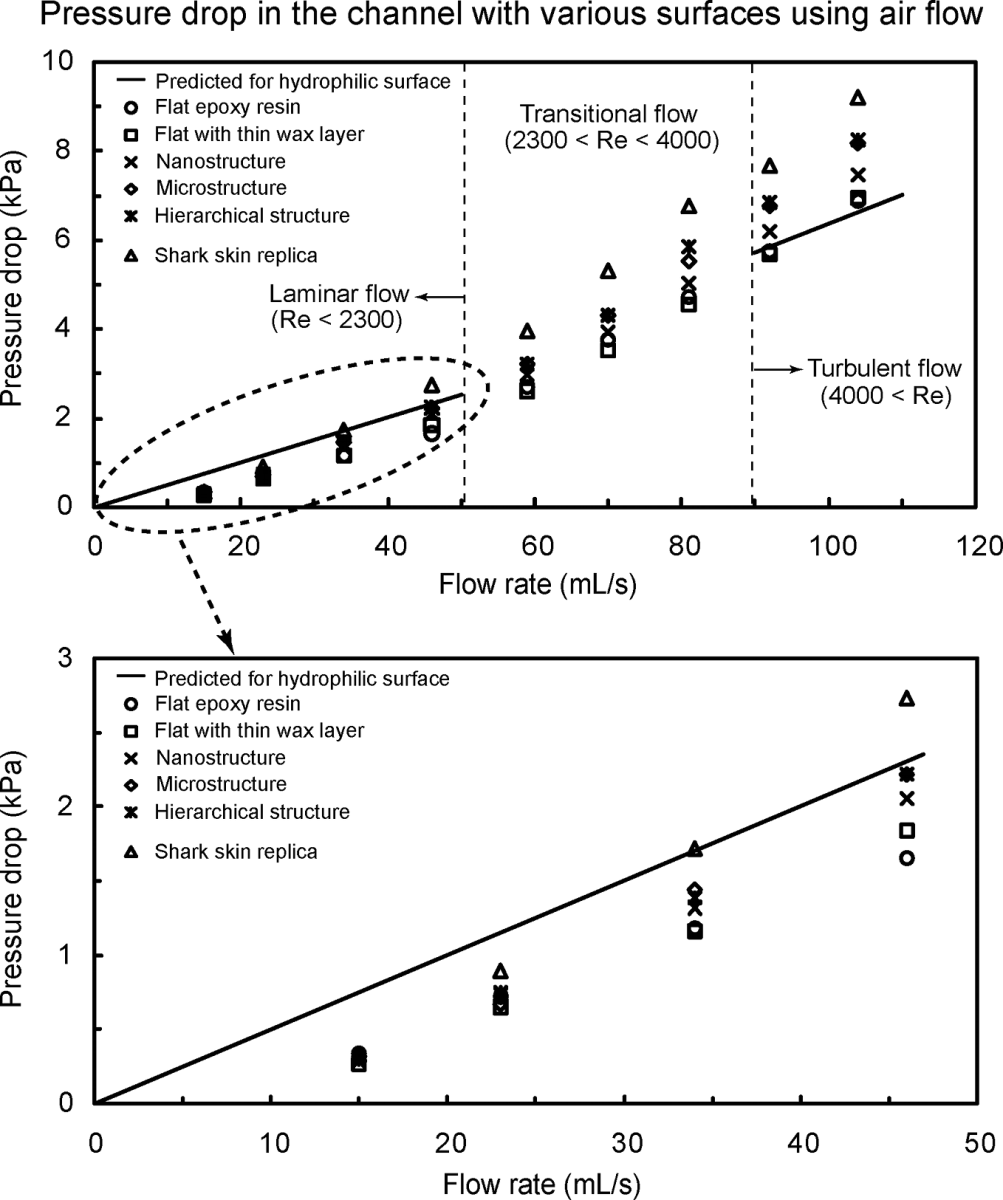
Pressure drop as a function of flow rate in the channel with various surfaces using air flow. The figure at the bottom is magnified for flow rate between 0 and 50 mL/s. Data are compared with predicted pressure drop values for a hydrophilic surface obtained using Equation 1 for laminar and turbulent flows (solid lines) [21].

**Pressure drop in the channel using air flow.** To investigate the effect of air flow in the channel and compare them to water drag reduction, experiments with air flow on various surfaces were performed [21]. In Figure 3, the rectangular channels had thickness *H* = 0.7 mm, width *W* = 2.5 mm, and length *L* = 60 mm. For calculation of pressure drop using Equation 1, the mass density ρ and viscosity η for air were taken to be 1.204 kg/m^3^ and 1.837×10^−5^ Pa·s, respectively [58]. Figure 9 shows the pressure drop as a function of flow rate in the channel with various surfaces using air flow. The measured data are compared with predicted pressure drop values for a hydrophilic surface obtained using Equation 1 for laminar and turbulent flows (solid lines). The figure at the bottom is magnified for flow rate between 0 and 50 mL/s. The pressure drop of the structured surfaces is higher than that of the hydrophilic surface in the turbulent flow which is opposite to that in liquid flow. In both laminar and turbulent flows, the pressure drop increased linearly with flow rate for all samples. As mentioned earlier, in the case of water flow, air pockets between the structures reduce the contact area between liquid and surface, resulting in reduction of the flow drag. The data shows that the structures are not beneficial for drag reduction in air flow. The introduction of roughness on the surfaces increases the pressure drop in the channel in the turbulent flow. It is generally known that surfaces with a streamlined body can produce dramatic reductions of the fluid pressure drag with only a slight increase in shear stress in air flow [60]. It is also known that as the Reynolds number increases, the pressure drop becomes very large, resulting in larger pressure drag. The roughness of structures on surfaces may cause air to move around them, resulting in the formation of vortices and large fluid drag.

**Figure 10:**
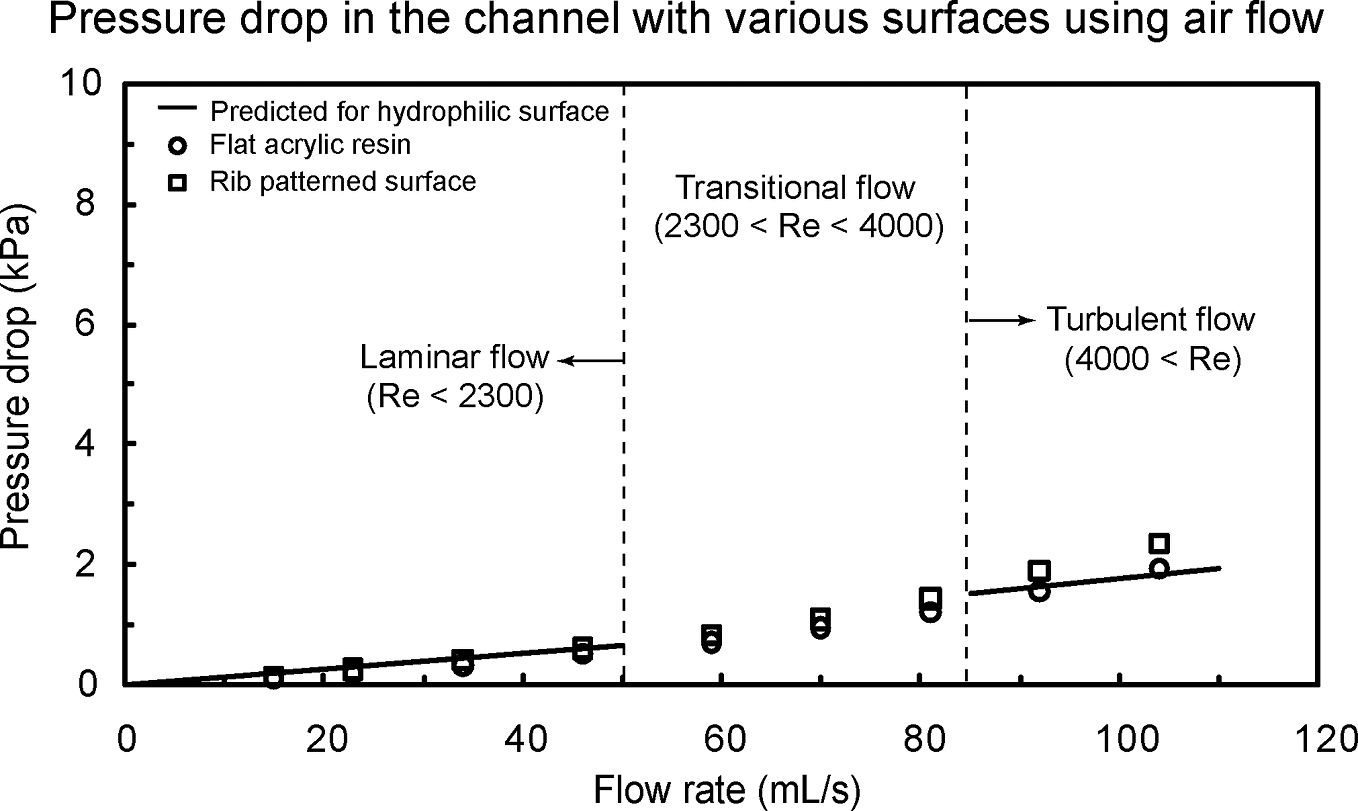
Pressure drop as a function of flow rate in the channel with flat acrylic resin and rib-patterned surface using air flow. Data are compared with predicted pressure drop values for a hydrophilic surface obtained using Equation 1 for laminar and turbulent flows (solid lines) [21].

To observe the fluid drag reduction in the channel using air flow, experiments on flat acrylic resin and fabricated rib-patterned surface were also performed [21]. The rectangular channels with these surfaces had thickness *H* = 1 mm, width *W* = 2 mm, and length *L* = 100 mm. Figure 10 shows the pressure drop as a function of flow rate in the channel with flat acrylic resin and rib-patterned surface using air flow. The measured data are compared with predicted pressure drop values for a hydrophilic surface obtained using Equation 1 for laminar and turbulent flows (solid lines). The experimental results show a similar trend to the data as shown in Figure 9. It was found that the pressure drop of the rib-patterned surface slightly increased due to the vortices formed at the end of the ribs in turbulent flow as compared to the theoretical pressure drop.

## Modeling, Fabrication and Characterization of Oleophobic/philic Surfaces

Oleophobic surfaces have the potential for self-cleaning and anti-fouling from biological and organic contaminants both in air and underwater applications. In this section, we discuss a model for predicting the oleophobic/philic nature and experimental measurements of the wetting properties of the surfaces.

### Modeling of contact angle for various interfaces

If a water droplet is placed on a solid surface in air, the solid–air and water–air interfaces come together with a static contact angle, θ_W_. The value of θ_W_ can be determined from the condition of the total energy of the system being minimized [61–63] and is given by Young’s equation for the contact angle θ_W_

[9]
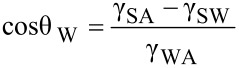


where γ_SW_, γ_SA_, and γ_WA_ are the surface tensions of the solid–water, solid–air, and water–air interfaces, respectively. If an oil droplet is placed on a solid surface in air, the Young’s equation for the contact angle, θ_O_, can be expressed by

[10]
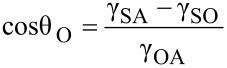


where γ_SO_, γ_SA_, and γ_OA_ are surface tensions of the solid–oil, solid–air, and oil–air interfaces, respectively. As predicted by Equation 10, if γ_SO_ is higher than γ_SA_, an oleophobic surface can be achieved.

To create an oleophobic surface in water, let us consider the solid–water–oil interface. If an oil droplet is placed on a solid surface in water, the contact angle of an oil droplet in water, θ_ΟW_, is given by Young’s equation

[11]
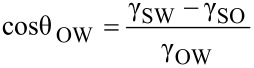


where γ_SO_, γ_SW_, and γ_OW_ are surface tensions of the solid–oil, solid–water, and oil–water interfaces, respectively. Combining Equation 9, Equation 10 and Equation 11, the equation for the contact angle, θ_OW_, of an oil droplet in water is given as

[12]
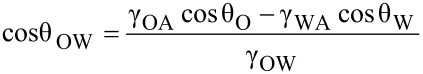


**Figure 11:**
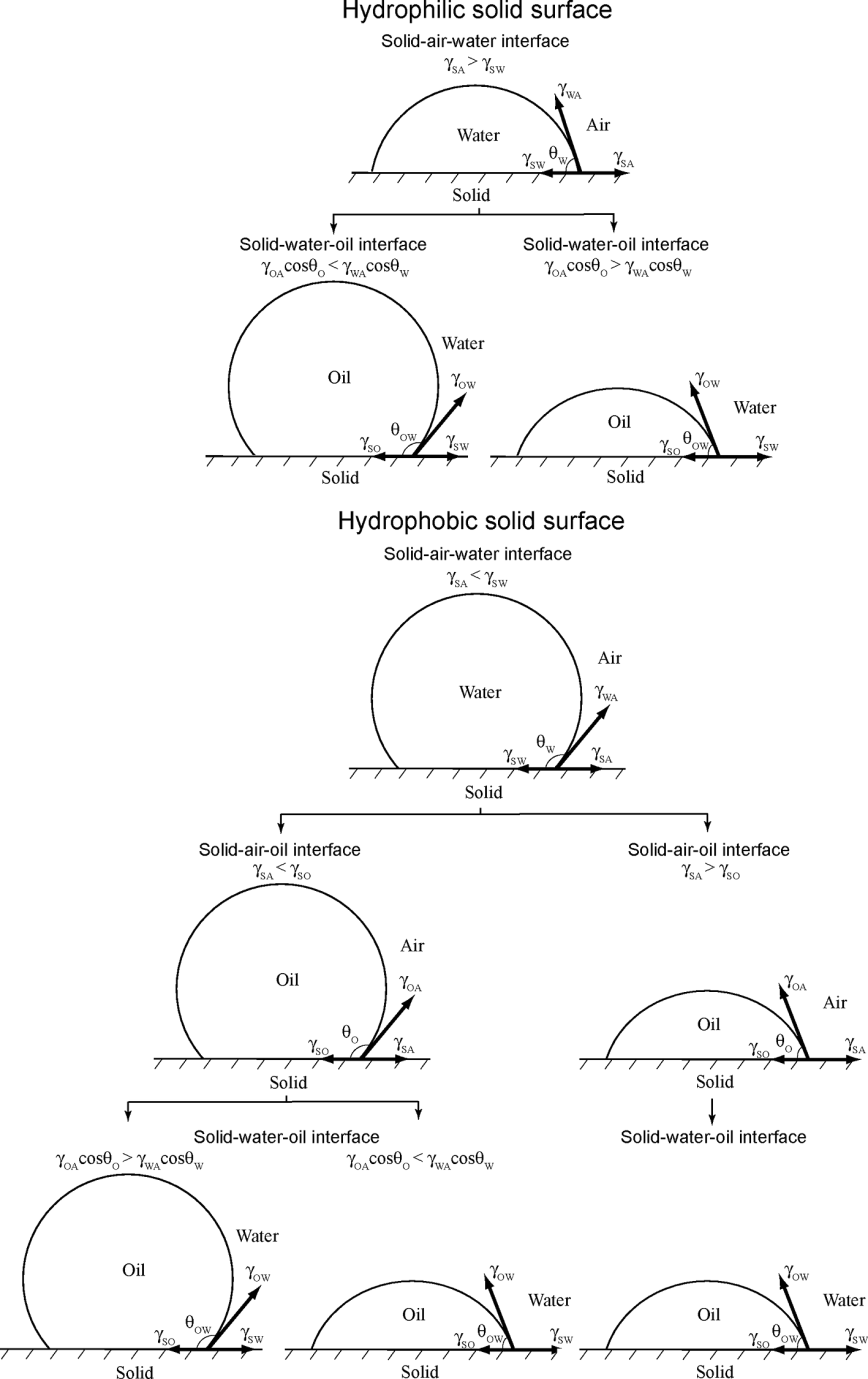
Schematics of a droplet of liquid showing philic/phobic nature in three different phase interface on the surface – θ_W_, θ_O_, and θ_OW_ are the static contact angles of a water droplet, an oil droplet, and an oil droplet in water, respectively [20].

Based on Jung and Bhushan [20], as predicted by Equation 12, for a hydrophilic surface (γ_SA_ > γ_SW_), an oleophobic surface in the solid–water–oil interface can be created if γ_OA_·cos θ_O_ is lower than γ_WA_*·*cos θ_W_. Since the surface tension of oil and organic liquids is much lower than that of water, most hydrophilic surfaces can be made oleophobic in a solid–water–oil interface. For a hydrophobic surface (γ_SA_ < γ_SW_) and an oleophobic surface in a solid–air–oil interface (γ_SA_ < γ_SO_), an oleophobic surface in a solid–water–oil interface can be created if γ_OA_·cos θ_O_ is higher than γ_WA_*·*cos θ_W_ and vice versa. For a hydrophobic and an oleophilic surface in solid–air–oil interface, an oleophobic surface in solid–water–oil interface cannot be created. Schematics are shown in Figure 11, and the summary of philic/phobic nature in various interfaces is shown in Table 3. For an oleophobic surface, oil contaminants are washed away when immersed in water. This effect leads to self-cleaning that can be used against ship fouling.

**Table 3:** Summary of philic/phobic nature in various interfaces [20].

**solid–air–water interface**		**solid–water–oil interface**

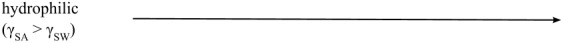		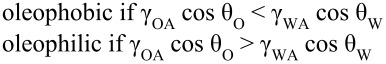

**solid–air–water interface**	**solid–air–oil interface**	**solid–air–water interface**

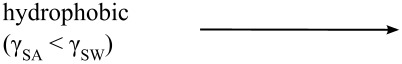	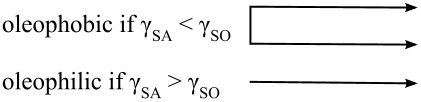	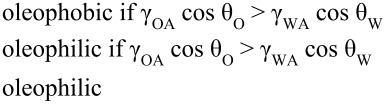

### Experimental techniques

**Figure 12:**
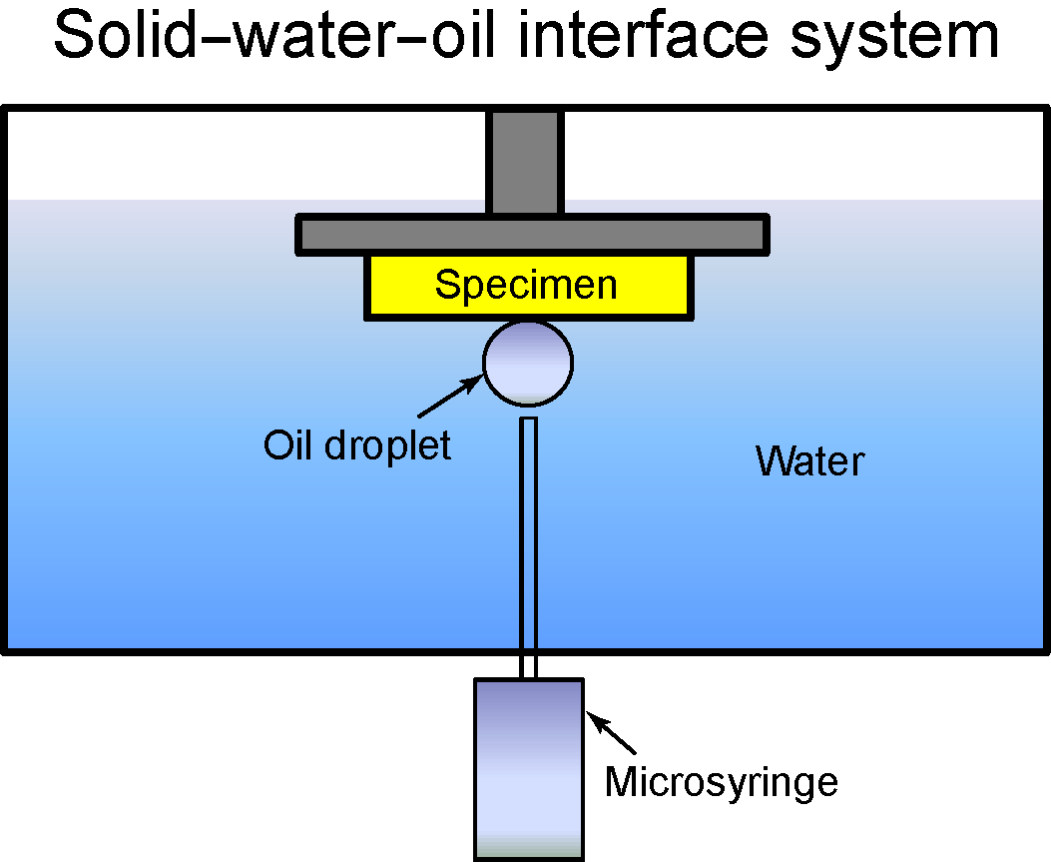
Schematics of a solid–water–oil interface system. A specimen is first immersed in water phase, then an oil droplet is gently deposited using a microsyringe, and the static contact angle in the system measured [20].

For the measurement of the static contact angle, deionized water was used for the water droplet and hexadecane was used for oil droplets [20]. The surface tensions of the water–air interface (γ_WA_), oil–air interface (γ_OA_), and oil–water interface (γ_OW_) are 73 [58], 27.5 [58], and 51.4 [64] mN/m, respectively. The mass densities are 1000 and 773 kg/m^3^ for water and hexadecane, respectively. Water and oil droplets of about 5 µL in volume (with radius of a spherical droplet about 1 mm) in an air environment were gently deposited on the specimen using a microsyringe. The process of wetting behavior of an oil droplet in water was obtained in a solid–water–oil interface system as shown in Figure 12 [20]. A specimen was first immersed in water phase. Then an oil droplet was gently deposited using a microsyringe from the bottom of the system because the density of oil (hexadecane) is lower than that of water. The image of the droplet was obtained by a digital camcorder (Sony, DCRSR100, Tokyo, Japan) with a 10× optical and 120× digital zoom. Images obtained were analyzed for the contact angle using Imagetool® software (University of Texas Health Science Center). The measurements were reproducible to within ± 2°.

### Fabrication and characterization of oleophobic surfaces

As presented earlier, a two-step molding process was used to replicate microstructures with varying pitch values. As a master template for flat and micropatterned surfaces, a flat Si surface and micropatterned Si surfaces with pillars of 14 μm diameter and 30 μm height with different pitch values (21, 23, 26, 35, 70, 105, 126, 168 and 210 μm), fabricated by photolithography, were used [20].

To study surfaces with some oleophobicity, a surface coating which has a lower surface tension than that of oil is required. For this purpose, Jung and Bhushan [20] deposited *n*-perfluoroeicosane (C_20_F_42_) (268828, Sigma-Aldrich, USA) on the specimen surfaces by thermal evaporation. The surface energy of *n*-perfluoroeicosane is 6.7 mJ/m^2^ (6.7 mN/m) [65]. The specimens were mounted on a specimen holder with double-sided tape and placed in a vacuum chamber at 30 mTorr (4 kPa pressure), 2 cm above a heating plate loaded with 6000 μg *n*-perfluoroeicosane [16]. The *n*-perfluoroeicosane was evaporated by heating it to 170 °C. In a vacuum chamber the evaporation from the point source to the substrate occurs in straight line; thus, the amount of sublimated material is equal in a hemispherical region over the point of source [66]. In order to estimate the amount of sublimated mass, the surface area of the half sphere was calculated by using the formula 2π*r*^2^, whereby the radius *r* represents the distance between the specimen to be covered and the heating plate with the substance to be evaporated. The calculated amount of *n*-perfluoroeicosane deposited on the surfaces was 2.4 µg/mm^2^ (amount of *n*-perfluoroeicosane loaded on a heating plate divided by surface area).

Hierarchical structures were fabricated using a two step fabrication process, including the production of microstructured surfaces by soft lithography and the subsequent development of nanostructures on top by self assembly of *n*-hexatriacontane with the amounts of 0.2 μg/mm^2^ deposited by thermal evaporation, as described previously [16,67]. Jung and Bhushan [20] also used a shark skin replica described previously.

**Figure 13:**
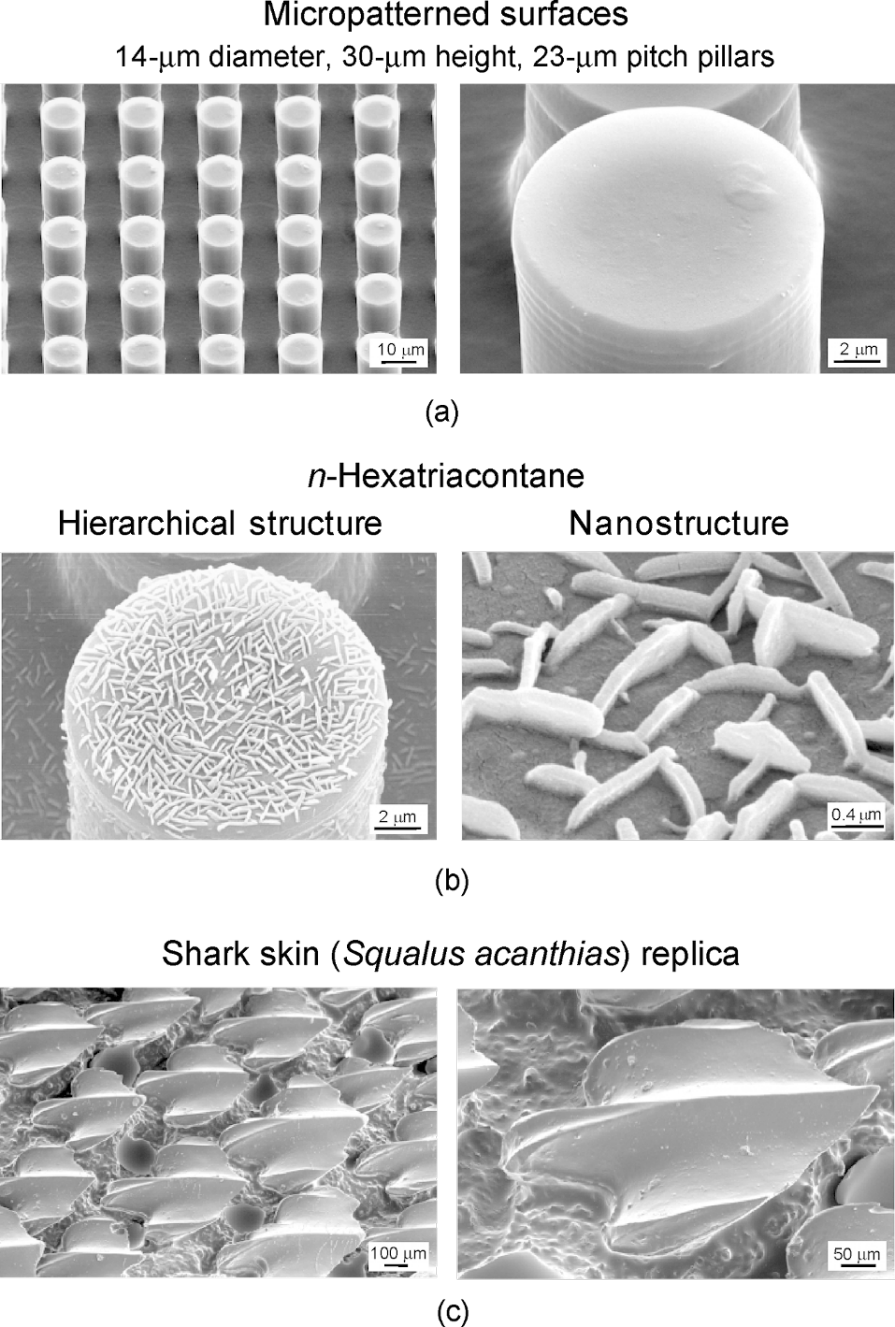
SEM micrographs taken at a 45° tilt angle showing two magnifications of (a) the micropatterned surface, (b) hierarchical structure and nanostructure with three-dimensional platelets on the surface fabricated with 0.2 µg/mm^2^ mass of *n*-hexatriacontane, and (c) shark skin (*Squalus acanthias*) replica. [20].

Figure 13a shows the SEM micrographs taken at a 45° tilt angle, showing two magnifications of the micropatterned surface. Figure 13b shows the hierarchical structures and nanostructures covered with *n*-hexatriacontane platelets. The nanostructure is formed by three-dimensional platelets of *n*-hexatriacontane. Platelets are flat crystals, grown perpendicular to the substrate surface. The platelet thickness varied between 50 and 100 nm, and their length varied between 500 and 1000 nm. Figure 13c, the shark skin replica, shows only three ribs on each scale. It is clearly visible that the V-shaped riblets’ height varies between 200 and 500 µm, and their space varies between 100 and 300 µm [20].

**Figure 14:**
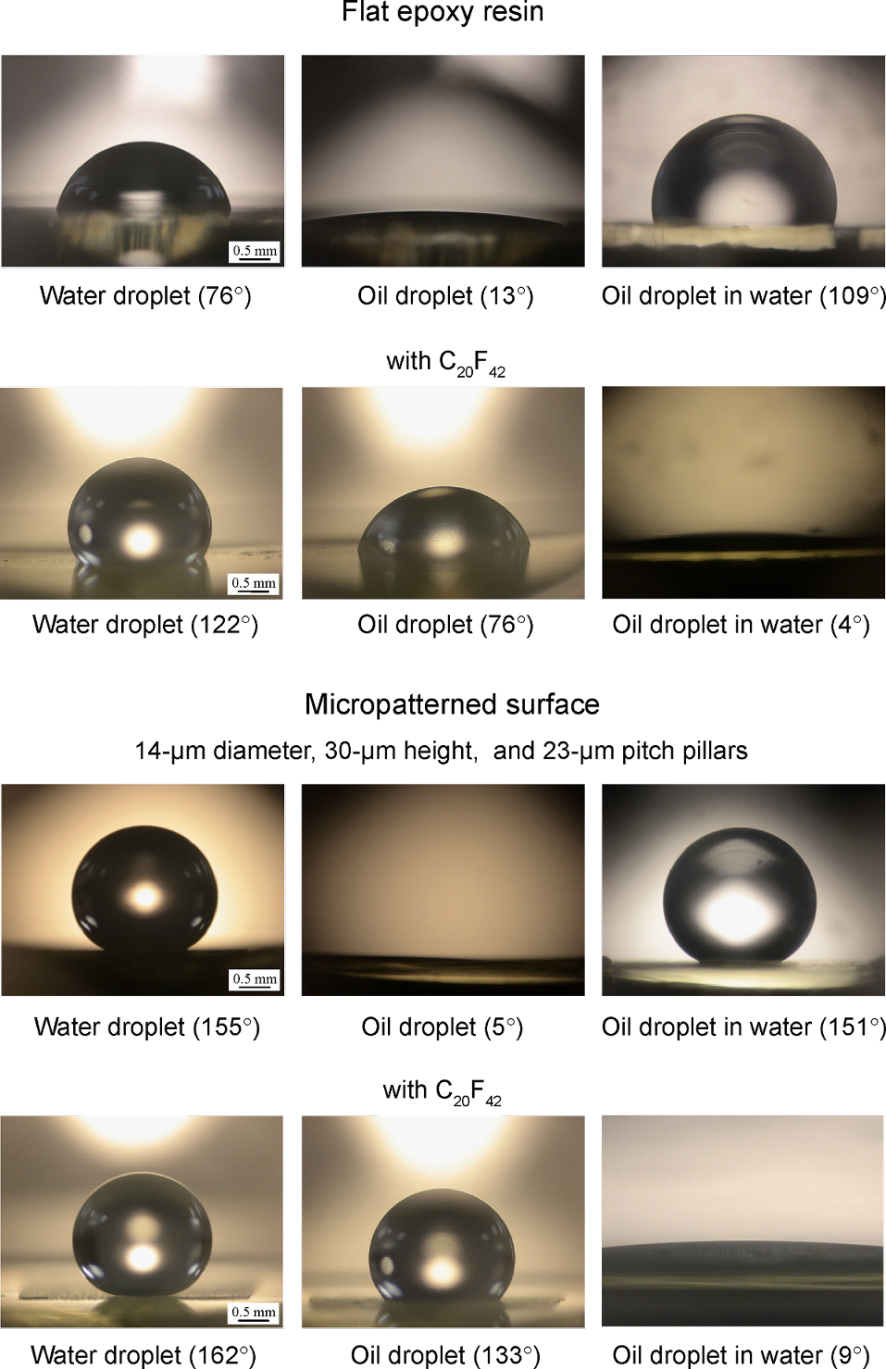
Optical micrographs of droplets in three different phase interfaces on flat epoxy resin and micropatterned surface without and with C_20_F_42_. Left images: a water droplet is placed on a surface in air. Middle images: an oil droplet is placed on a surface in air. Right images: an oil droplet is placed on a solid surface in water [20].

**Wetting behavior on flat and micropatterned surfaces.** To observe the wetting behavior of water and oil droplets for philic/phobic nature in three phase interfaces, Jung and Bhushan [20] performed experiments with droplets on both hydrophilic and hydrophobic, and oleophilic surfaces in air. Figure 14 shows the optical micrographs of droplets in three different phase interfaces on flat epoxy resin and micropatterned surfaces. In a solid–air–water interface, the water droplet was hydrophilic for the flat epoxy resin and was superhydrophobic for the micropatterned surface with 23 μm pitch. It is known that air pocket formation between the pillars makes a high static contact angle for micropatterned surface. However, in a solid–air–oil interface, the oil droplet was oleophilic for both surfaces. In the solid–water–oil interface system, in which the oil droplet sits on water trapped in the pillars, it is observed that the oil droplet in water was oleophobic and had contact angles of 109° and 151° for flat epoxy resin and micropatterned surface with 23 µm pitch, respectively.

**Figure 15:**
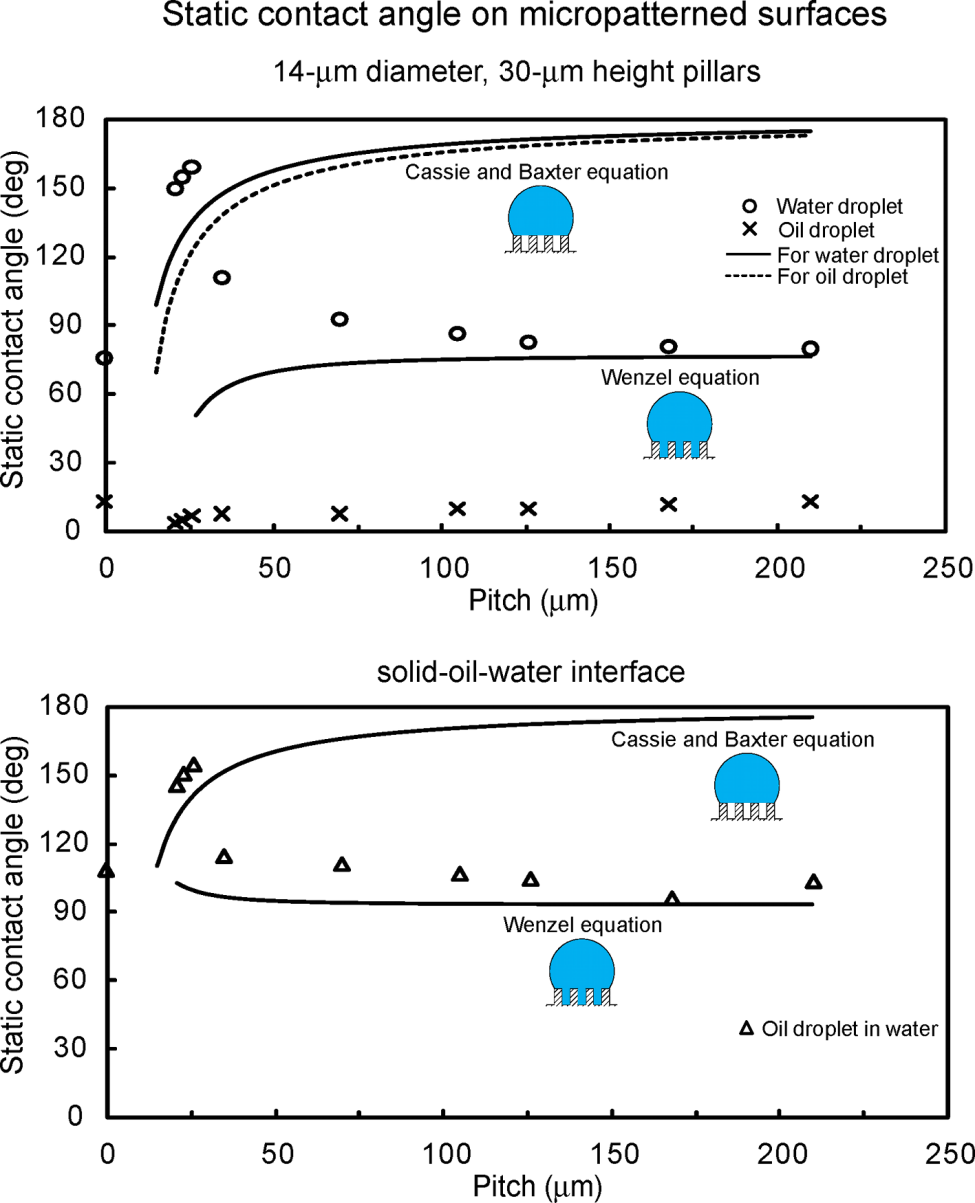
Static contact angle as a function of geometric parameters for water droplet (circle) and oil droplet (cross) in air (top), and oil droplet in water (triangle) (bottom) compared with predicted static contact angle values obtained using Wenzel and Cassie–Baxter equations (solid lines) with a measured value of θ_0_ for the micropatterned surfaces [20].

To study optimization of oleophobicity in the two solid–air–water and solid–air–oil interfaces, the static contact angles for water and oil droplets were measured on the micropatterned surfaces [20]. Figure 15 (top) shows the measured static contact angle as a function of pitch between the pillars for a water droplet (circle) and an oil droplet (cross) in air. The data are compared with predicted static contact angle values obtained using Wenzel and Cassie–Baxter equations [20] (solid lines) with a measured value of θ_0_ for the micropatterned surfaces. In a solid-air-water interface for a water droplet, the flat epoxy resin showed a static contact angle of 76°. The static contact angle on micropatterned surfaces is higher than that of the flat surfaces. It first increases with an increase in the pitch values, then starts to drop rapidly to a value slightly higher than that of the flat surfaces. In the first portion, it jumps to a high value of 150° corresponding to a superhydrophobic surface and continues to increase to 160° at a pitch of 26 μm because open air space increases with an increase in pitch responsible for the propensity of air pocket formation. The sudden drop at a pitch value of about 30 μm corresponds to the transition from the Cassie–Baxter to the Wenzel regime. The experimental observations for the transition are comparable to the value predicted from Wenzel and Cassie–Baxter equations.

At a solid–air–oil interface for an oil droplet, the flat epoxy resin showed a static contact angle of 13°. As shown in Figure 15 (top), the oil droplets on all micropatterned surfaces were oleophilic, and the contact angle was lower than that of the flat surfaces. It increases with an increase in the pitch values as predicted from Wenzel equation. As mentioned earlier, the surface tension of the oil–air interface is very low for hexadecane. Therefore, it is observed that from Equation 11 the surface tension of solid–oil interface (γ_SO_) is lower than that of solid–water interface (γ_SW_), resulting in oleophilic state for all micropatterned surfaces.

To study optimization of oleophobicity in a solid–water–oil interface, the static contact angles for oil droplets in water were measured on the micropatterned surfaces [20]. Figure 15 (bottom) shows the measured static contact angle as a function of pitch between the pillars for an oil droplet in water (triangles). The data are compared with the predicted static contact angle values obtained using the Wenzel and Cassie–Baxter equations [9] (solid lines), with a measured value of θ_0_ for the micropatterned surfaces. In a solid–water–oil interface, the oil droplet on the flat epoxy resin was oleophobic and had a static contact angle of 109°. The static contact angle of micropatterned surfaces in the solid–water–oil interface showed a similar trend to that in the solid–air–water interface. As the pitch increases up to 26 µm, the static contact angle first increases gradually from 146° to 155° because the oil droplet sits on water trapped in the pillars, and open space increases with an increase in pitch. The contact angle then starts to decrease rapidly due to the transition from the Cassie–Baxter to the Wenzel regime. The experimental observations for the transition are comparable to the values predicted from Wenzel and Cassie–Baxter equations. The micropatterned surfaces studied here were either hydrophilic or hydrophobic and both were oleophilic. In the solid–water–oil interface, they were oleophobic. It is observed that the data are not consistent with the model for hydrophobic surfaces shown in Figure 11 and Table 3. However, hydrophilic surfaces became oleophobic in the solid–water–oil interface because γ_OA_·cos θ_O_ is higher than γ_WA_·cos θ_W_.

**Wetting behavior on flat and micropatterned surfaces with C_20_F_42_.** To study surfaces with some oleophobicity, *n*-perfluoroeicosane (C_20_F_42_), which has lower surface tension than that of oil, was deposited on the surfaces, and experiments with droplets on hydrophobic and both oleophilic and oleophobic surfaces in air were performed [20]. Figure 14 shows the optical micrographs of droplets in three different phase interfaces on a flat epoxy resin and a micropatterned surface with C_20_F_42_. In a solid–air–water interface and a solid–air–oil interface, the water droplet and oil droplet showed contact angles of 122° and 76° for the flat epoxy resin with C_20_F_42_ and contact angles of 162° and 133° for the micropatterned surface with 23 µm pitch with C_20_F_42_, respectively. However, in a solid–water–oil interface, the oil droplet in water was oleophilic and had contact angles of 4° and 9° for both surfaces, respectively. To explain why the oleophobic surfaces in air became oleophilic in water, the theoretical values for both surfaces were calculated using Equation 12. For the calculations, the surface tensions of the water–air interface (γ_WA_), oil–air interface (γ_OA_), and oil–water interface (γ_OW_) were taken to be 73, 27.5, and 51.4 mN/m, and the contact angles for water and oil droplets in air were the measured values. The theoretical values for the flat epoxy resin and the micropatterned surface with 23 µm pitch with C_20_F_42_ are 28° and 10°, respectively. These values are similar to those from the experiments. This indicates that the oleophobic surfaces become oleophilic in water.

**Figure 16:**
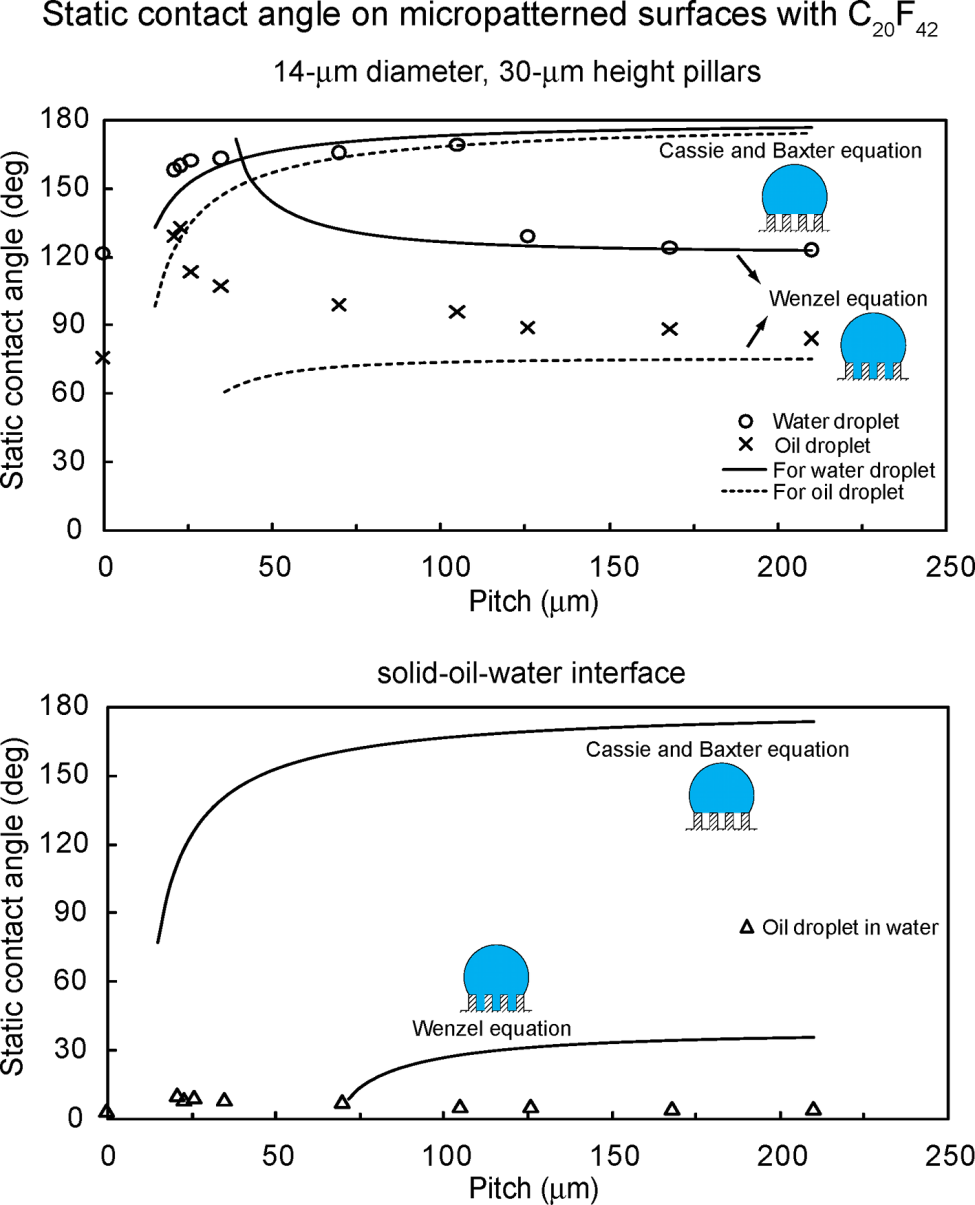
Static contact angle as a function of geometric parameters for water droplet (circle) and oil droplet (cross) in air, and oil droplet in water (triangles) compared with predicted static contact angle values obtained using the Wenzel and Cassie–Baxter equations (solid lines) with a measured value of θ_0_ for the micropatterned surfaces with C_20_F_42_ [20].

To study optimization of oleophobicity in two solid–air–water and solid–air–oil interfaces, the static contact angles for water and oil droplets were measured on the micropatterned surfaces with different pitch values and with C_20_F_42_ [20]. Figure 16 shows the measured static contact angle as a function of pitch between the pillars for a water droplet (circle) and an oil droplet (cross) in air. The data are compared with the predicted static contact angle values obtained using the Wenzel and Cassie–Baxter equations [20] (solid lines) with a measured value of θ_0_ for the micropatterned surfaces with C_20_F_42_. In a solid–air–water interface for the water droplet, the flat epoxy resin with C_20_F_42_ showed a static contact angle of 122°. The static contact angle of micropatterned surfaces with C_20_F_42_ first increases from 158° to 169° with an increase in the pitch values, then starts to drop rapidly at a pitch value of 110 µm. From a comparison of the experimental data with the Wenzel and Cassie–Baxter equations, this corresponds to the transition from the Cassie–Baxter to the Wenzel regime. All surfaces with C_20_F_42_ had an increase in contact angle, and the transition took place at higher pitch value than that of the micropatterned surfaces (Figure 15).

At a solid–air–oil interface for an oil droplet, the flat epoxy resin with C_20_F_42_ showed a static contact angle of 76°. As shown in Figure 16, the highest contact angle of micropatterned surfaces with C_20_F_42_ was 133° at a pitch value of 23 µm. Then, it decreases with an increase in the pitch values, and these values are comparable with the values predicted by the Wenzel equation. The contact angles of all micropatterned surfaces with C_20_F_42_ are higher than that of the flat surfaces.

To study optimization of oleophobicity in a solid–water–oil interface, the static contact angles for oil droplets in water were measured on the micropatterned surfaces with different pitch values and with C_20_F_42_ [20]. Figure 16 shows the measured static contact angle as a function of pitch between the pillars for an oil droplet in water (triangles). The data are compared with the predicted static contact angle values obtained using the Wenzel and Cassie–Baxter equations [20] (solid lines) with a measured value of θ_0_ for the micropatterned surfaces with C_20_F_42_. In a solid–water–oil interface, the flat epoxy resin with C_20_F_42_ was oleophilic and had a static contact angle of 4°. All micropatterned surfaces with C_20_F_42_ were oleophilic and had contact angle lower than 10°. The reason why hydrophobic and oleophobic surfaces in air became oleophilic in water can be explained from Figure 11 and Table 3. The contact angle for a water droplet is higher than that for an oil droplet on all surfaces with C_20_F_42_, and the surface tension of the water–air interface (γ_WA_) is higher than that of the oil–air interface (γ_OA_). Therefore, it is observed that γ_WA_·cos θ_W_ is higher than γ_OA_·cos θ_O_, and then the surfaces become oleophilic in the solid–water–oil interface.

**Figure 17:**
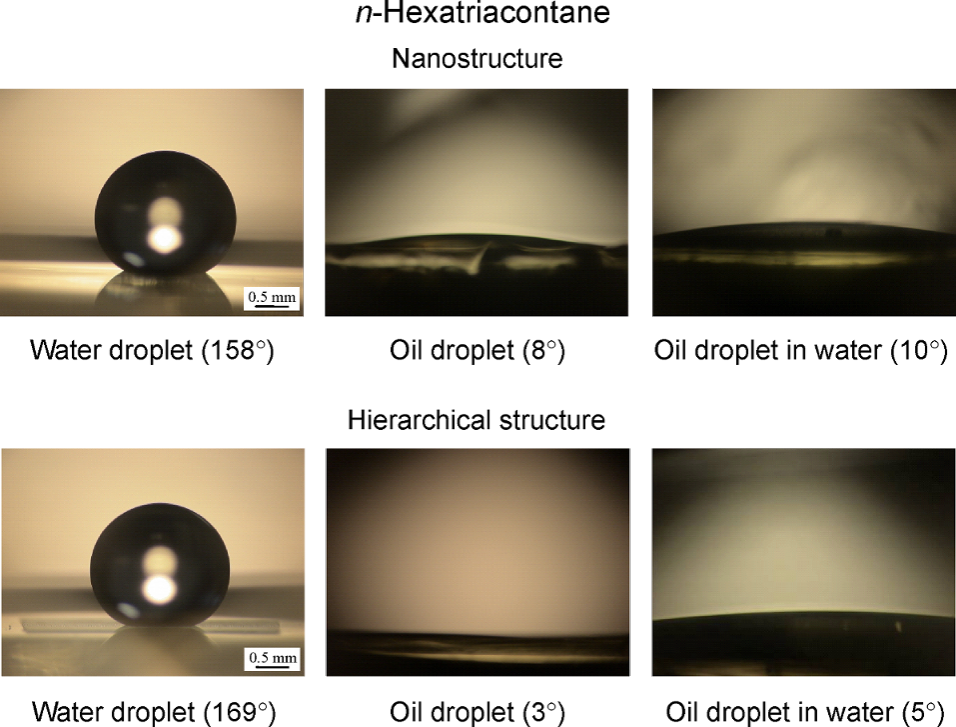
Optical micrographs of droplets in three different phase interfaces on nanostructure and hierarchical structure fabricated with 0.2 µg/mm^2^ mass of *n*-hexatriacontane. Left images: a water droplet is placed on a surface in air. Middle images: an oil droplet is placed on a surface in air. Right images: an oil droplet is placed on a solid surface in water [20].

**Wetting behavior on nano- and hierarchical structures and shark skin replica.** To observe the wetting behavior of water and oil droplets for nano- and hierarchical structures found from lotus plant surfaces, experiments with the droplets on the surfaces were performed in the three phase interface [20]. Figure 17 shows the optical micrographs of droplets in three different phase interfaces on a nanostructure and a hierarchical structure fabricated with 0.2 µg/mm^2^ mass of *n*-hexatriacontane. Both nano- and hierarchical structures were superhydrophobic and had a static contact angle of 158° and 169° in the solid–air–water interface, respectively. However, they are oleophilic in the solid–air–oil interface because the surface energy of *n*-hexatriacontane is 31.4 mJ/m^2^ (31.4 mN/m) [68], and this value is higher than that of an oil droplet (hexadecane). In the solid–water–oil interface, nano- and hierarchical structures had a static contact angle of 10° and 5°, respectively. As shown in Figure 11 and Table 3, it is observed that both surfaces are oleophilic in solid–water–oil interface.

**Figure 18:**
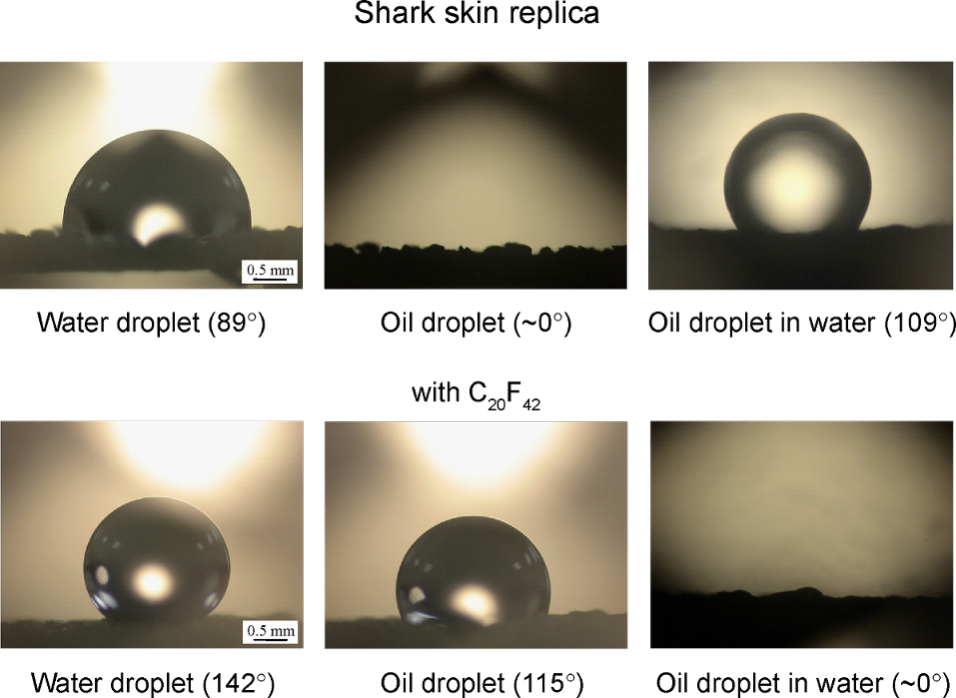
Optical micrographs of droplets in three different phase interfaces on shark skin replica without and with C_20_F_42_. Left images: a water droplet is placed on a surface in air. Middle images: an oil droplet is placed on a surface in air. Right images: an oil droplet is placed on a solid surface in water [20].

To study the surface structure of an aquatic animal, experiments with water and oil droplets on the shark skin replica were performed in a three phase interface [20]. Figure 18 shows the optical micrographs of droplets in three different phase interfaces on a shark skin replica without and with C_20_F_42_. First, the shark skin replica had contact angles of 89° and ~0° for water and oil droplets, respectively. After the surface was coated with C_20_F_42_, the contact angles of water and oil droplets became 142° and 115°, respectively. In the solid–water–oil interface, the oil droplet in water on the shark skin replica became oleophobic and had a contact angle of 109°. Based on Equation 12, the calculated value was 59° for the oil droplet in water on a shark skin replica. This difference may arise from the open space under the scales of the shark skin replica responsible for the propensity of trapped water pocket formation as reported by Jung and Bhushan [21]. Shark skin replica with C_20_F_42_ was oleophilic and had a contact angle of ~0°. This state is the same as the micropatterned surfaces with C_20_F_42_ as shown in Figure 11 and Table 3.

## Conclusion

Biomimetics allows one to mimic biology or nature and for engineers to develop materials and devices of commercial interest. Properties of biological materials and surfaces result from a complex interplay between surface morphology and physical and chemical properties. Hierarchical structures with dimensions of features ranging from the macroscale to the nanoscale are extremely common in nature and possess properties of interest. There are a large number of objects including bacteria, plants, land and aquatic animals and seashells, with properties of commercial interest.

One focus of this article is on biomimetics inspired structured surfaces for low fluid drag. One of the models from nature is the lotus leaf with a surface covered with wax and with hierarchical structure which provides superhydrophobicity, self cleaning, and low adhesion. An aquatic animal, such as a shark, is another model from nature. Shark skin is covered by very small individual tooth-like scales called dermal denticles (little skin teeth), ribbed with longitudinal grooves (aligned parallel to the local flow direction of the water). These grooved scales reduce vortices formation present on a smooth surface, resulting in water moving efficiently over their surface. The artificial surfaces inspired by the shark skin and the lotus leaf have been created and the influence of structure has been reviewed by measurement of pressure drop and fluid drag for drag reduction efficiency.

Oleophobic surfaces have the potential for self-cleaning and anti-fouling from biological and organic contaminants both in air and underwater applications. A model for predicting the contact angle of water and oil droplets has been reviewed. The surface tension of oil and organic liquids is lower than that of water, so to make the surface oleophobic in a solid–air–oil interface, a material with surface energy lower than that of oil should be used. The wetting behavior of water and oil droplets for hydrophobic/philic and oleophobic/philic surfaces in three phase interfaces is reviewed. For underwater applications, we have reviewed oleophobicity/philicity of an oil droplet in water on surfaces with different surface energies of various interfaces and contact angles of water and oil droplets in air.

This article provides a useful guide for the development of biomimetic artificial surfaces with either low drag or self-cleaning/anti-fouling properties.
